# Tobacco smoking is not associated with primary open-angle glaucoma: a systematic review and meta-analysis

**DOI:** 10.1007/s10792-026-03944-9

**Published:** 2026-02-02

**Authors:** Saajan Ramji, Sohail Daniel, Abdus Samad Ansari, Timothy L. Jackson, Abdulmalik Alsaif, Obeda Kailani

**Affiliations:** 1 North West London NHS Foundation Trust, London, UK; 2 Royal Bolton Hospital, Bolton NHS Foundation Trust, Bolton, UK; 3 King’s Ophthalmology Research Unit (KORU), Department of Ophthalmology, King’s College Hospital, London, UK; 4 Moorfields Eye Hospital NHS Foundation Trust, London, UK; 5 School of Life Course Sciences, Faculty of Life Science and Medicine, King’s College London, London, UK; 6 King Khaled Eye Specialist Hospital and Research Center (KKESH&RC), Riyadh, Saudi Arabia

**Keywords:** Smoking, Nicotine, Glaucoma, Systematic review, Meta-analysis

## Abstract

**Purpose:**

This systematic review explores the relationship between tobacco smoking and the development of primary open-angle glaucoma (POAG). Glaucoma is the leading cause of irreversible blindness worldwide. Prior literature investigating the link between tobacco smoking and glaucoma has reported contradictory findings on the association between tobacco smoking and POAG.

**Methods:**

Systematically EMBASE, MEDLINE, and Web of Science were searched, encompassing articles published up until April 2025. The inclusion criteria comprised observational and randomised controlled studies that provided a statistical analysis exploring the association between tobacco smoking and POAG in adult populations. The ROBINS-E tool was utilised to assess the risk of bias of studies and meta-analyses were completed using RevMan software. The main outcomes were effect estimates that measured the association between tobacco smoking and POAG.

**Results:**

Across 26 eligible studies and 289,930 participants, there was a prevalence of 6,454 cases of POAG. The meta-analyses revealed that current smokers (OR = 1.00, 95%CI 0.76–1.33, *p* = 0.97, n = 11), past smokers (OR = 0.92, 95%CI 0.75–1.11, *p* = 0.38, n = 6) as well as both current and past tobacco smoking combined (OR = 1.00, 95%CI 0.84–1.19, *p* = 1.00, n = 17) exhibited no statistically significant association with POAG when compared to individuals who had never smoked. Heterogeneity ranged from low to substantial across comparisons, and risk of bias was frequently rated as high among included observational studies. The lack of an associative effect was sustained, on exclusion of studies with a high risk of bias.

**Conclusion:**

Although the results suggest no significant statistical association between tobacco smoking and POAG, given the substantial morbidity and mortality associated with tobacco smoking and the detrimental impact on systemic and ocular health, tobacco smoking cessation should remain at the forefront of health promotion. PROSPERO registration ID: CRD42023409440.

## Introduction

Glaucoma encompasses a group of chronic diseases characterised by the progressive degeneration of the optic nerve and associated visual field loss [1]. It is the leading cause of irreversible sight loss and the second most common cause of blindness globally. Alarmingly, its global prevalence is projected to rise from 76 million in 2020 to 112 million by 2040 [2].

Primary open-angle glaucoma (POAG), the most common type of glaucoma often develops without symptoms until the later stages of the disease [3]. Elevated intraocular pressure (IOP) is considered a key modifiable risk factor for POAG [3, 4]. Raised IOP has been defined as a pressure exceeding 21 mmHg, with higher IOP levels linked to an increased risk of glaucomatous optic neuropathy [5]. Furthermore, evidence from pivotal trials within the field indicates that the risk of progression diminishes by 10% with each 1 mmHg reduction in IOP from the baseline [4].

The only modifiable risk factor with strong evidence for mitigating progression of primary open-angle glaucoma (POAG) is intraocular pressure (IOP) reduction. While lowering IOP slows disease progression, it does not address the underlying causes of disease onset, which remain incompletely understood. Established non-IOP-related epidemiological risk factors for POAG development include ethnicity, age, and corneal thickness [2, 5–11]. In efforts to better understand disease incidence and susceptibility, recent research has explored occupational and environmental influences. Among these, tobacco smoking has emerged as a potentially modifiable environmental exposure that may contribute to the development of POAG. Tobacco smoking remains highly prevalent, reported in 12.9% of the United Kingdom (UK) population and 32.6% of the global population [12, 13].

Tobacco smoking, as a modifiable risk factor, has been extensively researched and shown to be associated with various ocular diseases, such as age-related macular degeneration, age-related cataract, and thyroid eye disease [14–16].

To date, the research community has presented conflicting findings regarding the association between tobacco smoking and POAG. While some meta-analyses suggest that tobacco smoking increases the risk of POAG [17], others have found little evidence to support a causal link [18, 19]. Tobacco smoking has also been linked to elevated IOP [6, 20, 21], although the mechanism remains unclear.

Several biologically plausible mechanisms have been proposed to link tobacco smoking to POAG. One theory is that POAG may relate to reduced blood flow to the optic nerve head [22], and tobacco smoking contributes to vascular disease through atherosclerotic plaque formation, intimal thickening, and arterial lumen occlusion [23]. In addition, tobacco smoking induces oxidative stress through the production of free radicals, which may contribute to damage to the trabecular meshwork and retinal ganglion cells [24].

The most recent systematic review exploring the association between tobacco smoking and POAG [25] included studies published prior to February 2015, leading to the authors to conclude that evidence was limited for a link between tobacco smoking and glaucoma. Since this review, additional experimental studies have been published. The aim of this review is to provide a comprehensive update of the literature, to determine whether an association exists between tobacco smoking and the risk of POAG.

Tobacco smoking remains a significant public health concern, with approximately 1 billion smokers globally [26]. Should a definitive association between tobacco smoking and POAG be established, targeted health promotion and smoking cessation advice could potentially lower smoking prevalence and positively influence POAG rates. Such information would benefit policymakers and public health clinicians, aiding the implementation of public health measures to reduce smoking rates and providing an evidence base for offering smoking cessation advice in relation to this sight-threatening disease.

The aim of the study was to undergo a systematic review and meta-analysis with the following PICO model: In adults in population-based or clinical observational studies (P), is tobacco smoking exposure (current, former, or any smoking history, including dose measures such as pack-years where reported) (I), compared with never-smoking/non-smoking (C), associated with the incidence of primary open-angle glaucoma (O)?

## Methods

### Search methods for identifying studies

**Fig. 1:**
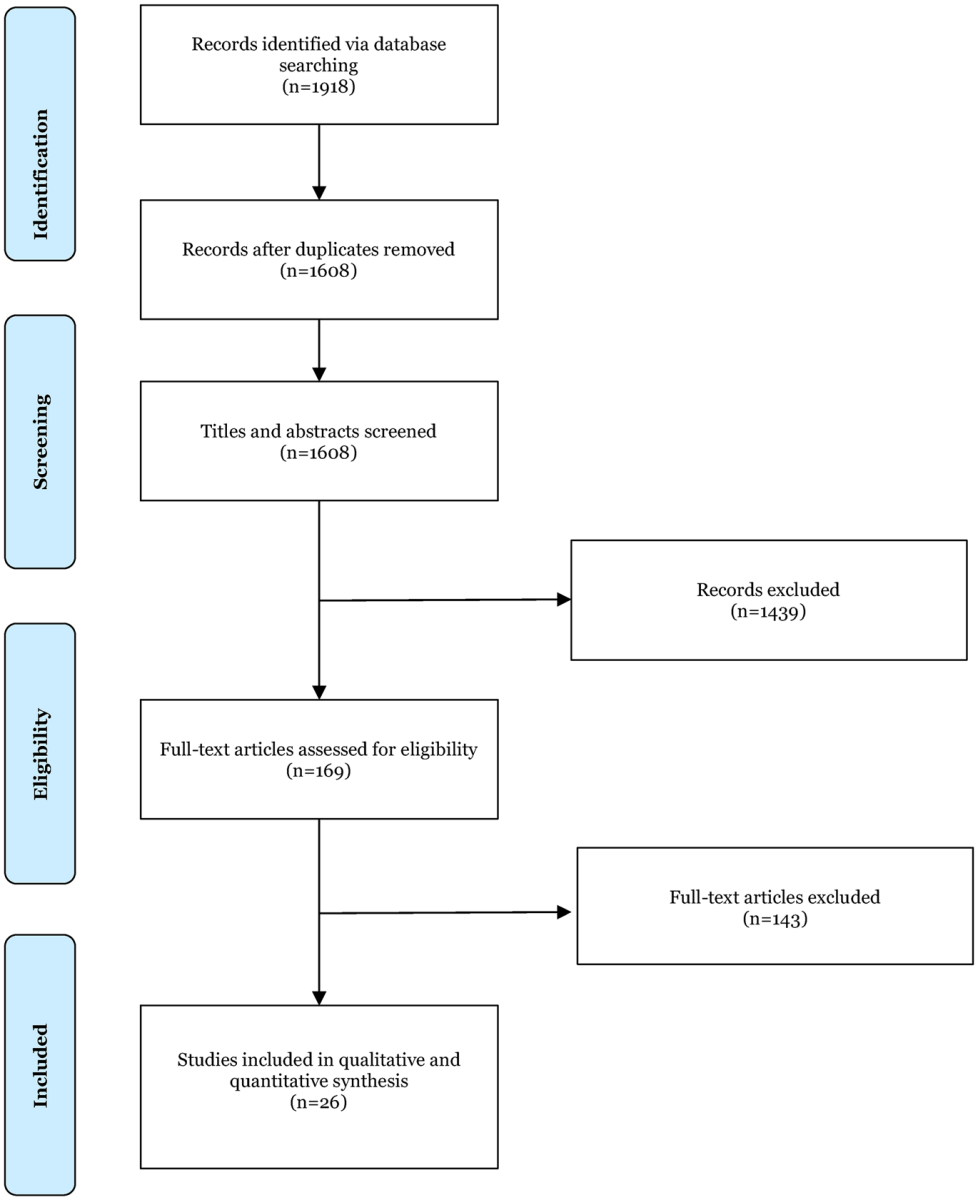
PRISMA flowchart of studies identified, screened and included

MEDLINE, EMBASE and Web of Science were systematically searched for studies published between 1st January 1946 and April 2025. The databases were searched from 1 January 1946 to April 2025 to ensure complete capture of the literature and continuity with earlier evidence. As the most recent comprehensive systematic review in this area included studies published up to February 2015 [25], our update focus was to identify and summarise evidence published from 1 January 2015 onwards; however, studies published before 2015 were screened against the same eligibility criteria and were included in qualitative synthesis and meta-analysis where extractable data permitted. The keywords and MESH headings used in the search included, ‘open-angle glaucoma,’ ‘intra-ocular pressure,’ or ‘intraocular pressure’ combined with, ‘smoking,’ ‘cigarette’ or ‘tobacco’. A full search strategy is presented in Fig. 1. Results from database searches and reference list screening were compiled onto Rayyan screening software [27]. The review was prospectively registered on PROSPERO (ID CRD42023409440).

### Eligibility criteria for considering studies for this review

Cohort, case–control, cross-sectional and randomised controlled trials that evaluated the association between cigarette smoking and POAG were included. Studies were required to report effect values (odds ratio [OR], risk ratio/relative risk [RR], hazard ratio [HR]) for the association between tobacco smoking and POAG. Studies with design types presenting level IV evidence or lower, such as letters, commentaries, case series, or case reports, were excluded [28]. Non-English language studies were excluded, unless there was an English translation available.

### Study selection

Studies that met the search criteria were exported into Rayyan software [27], and duplicates were removed. Screening was subsequently conducted by title and abstract. Following this, full articles were accessed, read, and evaluated against the inclusion and exclusion criteria. Once eligible studies were identified, reference lists were also screened for further studies. Both authors, SR and SD, performed the screening independently, and any disputes were resolved through discussion and consensus. If consensus could not be achieved, advice was sought from a third reviewer (OK).

### Data collection and risk of bias assessment

The collected data encompassed the author, country, study design, age, gender, cases of POAG, pack-year history, number of controls, the definition of smoking, and the adjustments or variables that were controlled for. Effect values were derived from each study, along with their 95% confidence intervals (95% CI) if available. If more than one effect value was presented, the value adjusted for the largest number of potential confounders was chosen. From each study, data was collected independently by SR and SD and input into a data collection table in Microsoft Excel [29]. Data were reviewed for accuracy. Any discrepancies were discussed and if consensus could not be established a third reviewer (OK) was consulted.

The ROBINS-E tool for assessing risk of bias in non-randomised studies was employed to evaluate the quality of the included studies by comparing them to an idealised target trial [30]. ROBINS-E is designed to be used in systematic reviews, allowing users to make both overall and specific judgements regarding the risk of bias (RoB) of the included studies across seven different criteria domains. Risk-of-bias assessment using ROBINS-E was performed independently by SR and SD; disagreements were resolved by discussion, with arbitration by a third reviewer (OK) if required. The GRADE rating system was used to assess the quality of effect estimates.

### Data synthesis and analysis

Effect values used for meta-analyses were ORs. Studies were eligible for pooling if they reported an OR (or an effect estimate that could be validly transformed) with an accompanying measure of variance (e.g., 95% CI or SE) and used a never-smoker comparator group. Studies that met inclusion criteria but did not report extractable variance (e.g., RR/IRR/HR without 95% CI, or non-combinable estimates such as β coefficients) were retained in the qualitative synthesis but were not included in the meta-analysis. RevMan (Cochrane Review Manager version 5.4) software was used to convert effect values to their natural logarithm and to calculate standard error (SE) from 95% CIs, which were then used for pooled analysis. The three groups where meta-analysis was conducted compared non-smokers (controls) to: current smokers, past smokers and current & past smokers combined (studies not differentiating between past and current). A final meta-analysis of all studies reporting any smoking history was conducted. A *p*-value < 0.05 was considered statistically significant for all statistical tests. Meta-analyses were performed in RevMan using the software’s default inverse-variance fixed- and random-effects models; no additional bespoke statistician-led analyses were undertaken.

Given anticipated clinical and methodological heterogeneity across observational studies (including variation in smoking exposure definitions, study design, populations, and covariate adjustment), we interpret pooled estimates primarily under a random-effects framework when heterogeneity is present, representing an average effect across study settings. Heterogeneity was assessed using Cochrane’s Q test and the I^2^ statistic. To assess the robustness of the pooled estimates, exclusion-based sensitivity analyses for each main outcome were performed by removing studies judged to be at high overall risk of bias using the ROBINS E tool.

## Results

### Search results

The search yielded 1,918 articles. Following duplicate removal 1,608 remained, of which 1,439 were excluded during the title and abstract screening stage. The remaining 169 articles underwent full text screening; of these, 143 articles were excluded, resulting in 26 eligible articles. Zhou et al. [53] and a study by Buys et al. (2012) had the same results and therefore were deemed to be duplicates. Consequently, the paper which first published the results was included in our review and meta-analysis. Figure 1 shows the PRISMA flow chart, detailing study selection.

### Study characteristics

The 26 eligible studies included 289,930 participants with 6,454 cases of POAG (Tables 1, 2, 3, 4). Of the 26 studies, seven were cohort, fourteen were case–control and five were cross-sectional (Tables 1, 2, 3, 4). A total of eleven studies were conducted in the USA, three in China, two in Canada, two in Iran, two in Australia, two in Congo, one in France, one in Spain, one in Singapore, and one in Serbia (Tables 1, 2, 3, 4). The control groups included 5,261 current smokers, 5,931 past smokers and 248 participants from studies with non-specific smoking history, grouping study groups into a ‘current and past smokers’ cohort (Tables 1, 2, 3, 4). In the POAG groups, there were 707 current smokers, 1,225 past smokers and 235 who were grouped into current and past smokers (Tables 1, 2, 3, 4).

**Table 1:** Baseline characteristics and effect estimates of all included studies that incorporated current smokers

Study ID (Author, location and year of publication)	Type of study	Total Number of Patients			Definition of smokers	Control Group Selection	Control Group	POAG Group	Adjustments & variables controlled for in study	Current Smoking Odds Ratio (95% CI)	Current Smoking Relative Risk or Risk Ratio Unless Specified (95% CI)
		Total Participants (M/F)	Control (M/F)	POAG (M/F)			Current smokers (%)	Current smokers (%)			
*Cohort Studies*											
Kang et al., USA [36]	Prospective cohort	71,819 (28,139/ 43,680)	71,369 (N/A)	450 (N/A)	Smokers defined as current, past and non-smokers. No further definitions given	Controls were drawn from two large US cohorts (NHS and HPFS) of health professionals aged ≥ 40 years, free of glaucoma at baseline, and who reported undergoing eye examinations during follow-up. Eligibility was updated biennially. The cohorts were predominantly white, and data were collected through validated, repeated questionnaires	–	48 (10.7)	Age, sex, BMI, hypertension, diabetes, ethnicity and alcohol intake	-	0.85 (0.62 – 1.18) no p-value
Doshi et al., USA [34]	Retrospective cohort	6142 (N/A)	5624 (N/A)	289 (N/A)	Smokers defined as current, past and non-smokers. No further definitions given	Controls were self-identified Latino participants aged ≥ 40 from six census tracts in La Puente, California, who completed both home interviews and in-clinic examinations and were found to have neither OAG nor OHT	802 (14.7)	21 (7.3)	Age and gender	0.59 (0.36 – 0.96) no p-value	-
Wise et al., USA [48]	Prospective cohort	32,570 (0/32,570)	32,204 (0/32204)	366 (0/366)	Smokers defined as current, past and non-smokers. No further definitions given	Controls were African-American women aged 21–69 from the Black Women’s Health Study, who completed follow-up questionnaires and did not develop POAG. Only those reporting recent eye exams were included to minimise detection bias. Selection was prospective, updated biennially, and based on physician-confirmed non-cases within the analytic cohort	-	49 (13.4)	Alcohol consumption, diabetes, BMI, age, ex, weight, height and education	-	Incidence rate ratio 0.97 (0.7 – 1.35) no p-value
Ramdas et al., Netherlands [43]	Prospective cohort	3939 (1638/2301)	3831 (1583/ 2248)	108 (55/53)	Smokers defined as current, past and non-smokers. No further definitions given	Controls were participants aged ≥ 55 years from the population-based Rotterdam Study, who were free of OAG at baseline and completed at least one follow-up examination. All underwent standardised ophthalmic exams and lifestyle assessments. Selection excluded those with glaucomatous visual field loss at baseline	1269 (33.4)	36 (33.3)	Age, gender, IOP at baseline & IOP lowering treatment	-	Male Beta -0.40 (0.49) P = 0.41 Female Beta -0.22 (0.21) P = 0.29
Perez-de-Arcelus et al., Spain [41]	Prospective cohort	16,797 (6683/10114)	16,613 (N/A)	184 (N/A)	Smokers defined as current, past and non-smokers. No further definitions given	Controls were university graduates from the SUN cohort who were free of glaucoma at baseline and had complete smoking data. Follow-up was via biennial questionnaires. Those with glaucoma at baseline or implausible energy intakes were excluded. Cohort included mainly Caucasian, highly educated individuals, with 50% being health professionals	-	82 (44.6)	Age, sex, BMI, hypertension, T2DM, physical activity, coffee consumption, alcohol, Mediterranean diet, total energy intake	-	Hazard ratio 1.88 (1.26 – 2.81) p = 0.002
*Case–control Studies*											
Morgan & Drance, Canada [40]	Case–control	182 (N/A)	91 (N/A)	91 (N/A)	Current smoker was individual who currently smoked > 20 cigarettes a day	Controls were community residents in British Columbia, matched to glaucoma patients by age (± 5 years) and sex. Selected by visiting the fourth house to the right of each case’s home. Few refusals; controls were similar to cases in education and income. Interviews were conducted in-home by a trained interviewer	12 (13.2)	10 (11)	Age, sex, education and income level	-	1.0 (No CI) no p-value, states not significant
Reynolds, USA [44]	Case–control	174 (N/A)	87 (N/A)	87 (N/A)	Current smoker, no further definitions	Controls were selected from general clinic patients at the same centre and time as glaucoma cases. Matched by age, sex, and race, with attempts to match socioeconomic status and refractive error. Selection used a random starting point, listing every third matched patient until pairs were completed	28 (32.2)	28 (32.2)	Sex, age and race	-	1.0 (No CI) no p-value, states not significant
Wilson et al., USA [47]	Case–control	320 (153/167)	237 (105/132)	83 (48/35)	Smokers were current smokers, no further definitions given	Controls were randomly selected from General Eye Service attendees at Massachusetts Eye and Ear Infirmary, matched to cases by sex and 5-year age categories. They underwent routine eye exams excluding visual fields. Interviewed in clinic using predefined selection rules. Only those without POAG or suspicion of POAG were included	55 (23.2)	24 (28.9)	Age, gender, race, hypertension, family history	-	Rate ratio 2.9 (1.3 – 6.6) no p-value
Katz & Sommer, USA [37]	Case–control	188 (102/86)	94 (51/43)	94 (51/43)	Smokers were current smokers and either smoked 20 cigarettes a day for 10 or more years, or 10–20 a day for 15 or more years	Controls were matched to glaucoma cases by age, race, and sex. All participants completed structured interviews on ocular/systemic history, medications, smoking, and alcohol. Controls had no documented glaucomatous visual field loss and were selected for direct comparison with confirmed glaucoma cases	31 (33.3)	24 (25.8)	Age, sex, ethnicity	0.7 (0.37 – 1.32) no p-value, states not significant	-
Charliat, Jolly & Blanchard, France [31]	Case–control	350 (N/A)	175 (N/A)	175 (N/A)	Smokers defined as current, past and non-smokers. No further definitions given	Controls were matched to cases by age (± 2 years), sex, and healthcare setting. All were Caucasian, ≥ 40 years, with normal IOP, optic discs, and visual fields. Recruited from private practices and public hospitals, controls were mostly outpatients undergoing routine vision checks, including visual fields even without glaucoma suspicion	24 (13.7)	28 (16)	Age, gender, type of hospital care	1.4 (0.7 – 2.77) no p-value, states not significant	-
Kaimbo & Missotten, Congo [35]	Case–control	260 (N/A)	238 (N/A)	22 (N/A)	Current smoker, no further definitions given	Controls were factory workers in Kinshasa, aged 24–60, without optic disc damage or glaucomatous visual field defects. Selected from the same workplace cohort as cases, all participants underwent interviews and ocular examinations	-	-	-	2.77 (1.04 – 7.34) no p-value	-
Wang et al., China [46]	Case control	248 (199/49)	126 (97/29)	*Total 122 (102/20) JOAG 30 AOAG 92	Smoker were defined as active smokers. They were defined as smoking 5 or more cigarettes a day for the past year or more	Controls were senile cataract surgical inpatients aged ≥ 50 years, without family history of glaucoma, IOP < 21 mmHg, VCDR < 0.5, and no visual field loss. Recruited from the same hospital as cases (Shantou, China), all underwent detailed ocular exams and were unrelated to POAG patients	71 (56.3)	Total = 77 (63.1) JOAG = 16 (58.5) AOAG = 61 (66.3)	Age & gender	*JOAG 4.33 (0.12 – 0.0000584) p = 0.692 AOAG 1.03 (0.52 – 2.08) p = 0.925	-
Charlson et al., USA [32]	Case–control	2067 (765/1302)	807 (265/542)	1260 (500/760)	Smokers defined as current, past and non-smokers. No further definitions given	Controls were African American adults aged > 35 recruited from ophthalmology clinics at the University of Pennsylvania. Excluded if they had high myopia, family history of POAG, abnormal visual fields, IOP > 21 mmHg, or glaucomatous optic nerve findings. All underwent standardised clinical exams and detailed phenotyping by glaucoma specialists	93 (13.7)	143 (12.3)	Age, race	1.42 (1.03 – 1.97) p = 0.07	-
Stamenkovic et al., Serbia [45]	Case–control	304 (136/168)	202 (91/111)	102 (45/57)	Smokers were defined as those that smoked every day for a minimum of 60 days before the enrolment in the study	Controls were 202 Caucasian individuals without POAG, matched to 102 POAG patients by age and gender. Recruited from Zvezdara University Medical Centre, Belgrade. Excluded if IOP > 21 mmHg, optic disc cupping, or glaucomatous visual field defects. All completed questionnaires and genotyping	66 (32.7)	47 (46.1)	Age, gender	2.00 (1.20 – 3.35) p = 0.008	-
Chiam et al., Singapore [33]	Case–control	3499 (1839/1660)	2788 (1361/ 1427)	711 (478/233)	Smokers defined as current, past and non-smokers. No further definitions given	Controls were Chinese Singaporeans aged ≥ 40 from the population-based Singapore Chinese Eye Study (2009–2011). Eligible controls had IOP ≤ 21 mmHg, open angles, healthy optic discs, and normal visual fields. Participants with glaucoma or other ocular diseases were excluded. All underwent comprehensive ophthalmic exams and interviews	382 (13.7)	65 (9.2)	States multiple logistic regression and multivariate analysis	0.45 (0.20 – 1.00) p = 0.05	-
*Cross-sectional Studies*											
Klein et al., USA [39]	Cross-sectional	4926 (N/A)	4822 (N/A)	104 (N/A)	Smoking less than 100 cigarettes in a lifetime was a non-smoker Those who had smoker above 100 and stopped were past smokers Current smokers were participants that currently smoked but no further definitions given	Controls were selected from a population-based cohort of adults aged 43–84 years in Beaver Dam, Wisconsin. Participants underwent standardized ophthalmic assessments including IOP measurement, optic disc photography, and visual field testing. Controls were individuals not meeting criteria for definite or probable POAG based on established diagnostic protocols	-	-	Does not specify	Men 0.74 (0.25 – 2.17) no p-value Women 1.11 (0.54 – 2.30) no p-value	-
Quigley et al., USA [42]	Cross-sectional	4768 (1849/2919)	4674 (1618/ 2858)	94 (33/61)	Smokers were categorised as current, past and non-smokers but no further definitions were given	Controls were Hispanic adults aged ≥ 40 years from randomly selected census block groups in Arizona. Participants completed home interviews and standardized clinic examinations, including IOP, optic disc, and visual field assessments. Controls were those not meeting international diagnostic criteria for POAG	965 (20.7)	9 (9.6)	Age and ethnicity	0.71 (0.35 – 1.48) no p-value	-
Khalili et al., Iran [38]	Cross-sectional	11,208 (4992/6216)	11,092 (4934/ 6158)	116 (58/58)	Non-smokers smoked < 100 cigarettes in lifetime, ex-smokers had smoked > 100 and stopped 1 year before study questionnaire, regular smokers smoked at least 1 cigarette a day and heavy smokers smoked > 20 cigarettes a day. Regular and heavy smokers are current smokers	Controls were adults aged 35–70 years from the Azar cohort (Iran), who completed comprehensive eye exams and did not meet diagnostic criteria for POAG. Selection was population-based. All underwent optometrist-led screening, with referrals for full ophthalmological assessment based on predefined clinical flags, including IOP > 21 mmHg and family history	Regular smoker 879 (7.9) Heavy smoker 584 (5.3)	Regular smoker 7 (6) Heavy smoker 9 (7.8)	Age	Regular smoker 0.61 (0.27 – 1.39) p = 0.245 Heavy smoker 1.17 (0.55 – 2.47) p = 0.671	-

**Table 2:** Baseline characteristics and effect estimates of all included studies that incorporated past smokers

Study ID (Author, location and year of publication)	Type of study	Total Patients			Definition of smokers	Control Group Selection	Control Group	POAG Group	Adjustments & variables controlled for in study	Past Smoking Odds Ratio (95% CI)	Past Smoking Relative Risk or Risk Ratio Unless Specified (95% CI)
		Total Participants (M/F)	Control (M/F)	POAG (M/F)			Past smokers (%)	Past smokers (%)			
*Cohort Studies*											
Kang et al., USA [36]	Prospective cohort	71,819 (28,139/ 43,680)	71,369 (N/A)	450 (N/A)	Smokers defined as current, past and non-smokers. No further definitions given	Controls were drawn from two large US cohorts (NHS and HPFS) of health professionals aged ≥ 40 years, free of glaucoma at baseline, and who reported undergoing eye examinations during follow-up. Eligibility was updated biennially. The cohorts were predominantly White, and data were collected through validated, repeated questionnaires	-	181 (40.2)	Age, sex, BMI, hypertension, diabetes, ethnicity and alcohol intake	-	0.91 (0.63 – 1.32) no p-value
Doshi et al., USA [34]	Retrospective cohort	6142 (N/A)	5624 (N/A)	289 (N/A)	Smokers defined as current, past and non-smokers. No further definitions given	Controls were self-identified Latino participants aged ≥ 40 from six census tracts in La Puente, California, who completed both home interviews and in-clinic examinations and were found to have neither OAG nor OHT	1349 (23.9)	86 (29.8)	Age and gender	0.89 (0.66 – 1.21) no p-value	-
Wise et al., USA [48]	Prospective cohort	32,570 (0/32,570)	32,204 (0/32204)	366 (0/366)	Smokers defined as current, past and non-smokers. No further definitions given	Controls were African-American women aged 21–69 from the Black Women’s Health Study, who completed follow-up questionnaires and did not develop POAG. Only those reporting recent eye exams were included to minimise detection bias. Selection was prospective, updated biennially, and based on physician-confirmed non-cases within the analytic cohort	-	116 (31.7)	Alcohol consumption, diabetes, BMI, age, ex, weight, height and education	-	Incidence rate ratio 0.89 (0.70 – 1.13) no p-value
Ramdas et al., Netherlands [43]	Prospective cohort	3939 (1638/2301)	3831 (1583/ 2248)	108 (55/53)	Smokers defined as current, past and non-smokers. No further definitions given	Controls were participants aged ≥ 55 years from the population-based Rotterdam Study, who were free of OAG at baseline and completed at least one follow-up examination. All underwent standardised ophthalmic exams and lifestyle assessments. Selection excluded those with glaucomatous visual field loss at baseline	1719 (45.3)	53 (49.1)	Age, gender, IOP at baseline & IOP lowering treatment	-	Male Beta -0.82 (0.38) P = 0.03 Female Beta 0.06 (0.16) P = 0.70
Perez-de-Arcelus et al., Spain [41]	Prospective cohort	16,797 (6683/10114)	16,613 (N/A)	184 (N/A)	Smokers defined as current, past and non-smokers. No further definitions given	Controls were university graduates from the SUN cohort who were free of glaucoma at baseline and had complete smoking data. Follow-up was via biennial questionnaires. Those with glaucoma at baseline or implausible energy intakes were excluded. Cohort included mainly Caucasian, highly educated individuals, with 50% being health professionals	-	49 (26.6)	Age, sex, BMI, hypertension, T2DM, physical activity, coffee consumption, alcohol, Mediterranean diet, total energy intake	-	Hazard ratio 1.27 (0.88 – 1.82) p = 0.198
*Case–control Studies*											
Charliat, Jolly & Blanchard, France [31]	Case–control	350 (N/A)	175 (N/A)	175 (N/A)	Smokers defined as current, past and non-smokers. No further definitions given	Controls were matched to cases by age (± 2 years), sex, and healthcare setting. All were Caucasian, ≥ 40 years, with normal IOP, optic discs, and visual fields. Recruited from private practices and public hospitals, controls were mostly outpatients undergoing routine vision checks, including visual fields even without glaucoma suspicion	48 (27.4)	52 (29.7)	Age, gender, type of hospital care	1.34 (0.72 – 2.48) no p-value states not significant	-
Charlson et al., USA [32]	Case–control	2067 (765/1302)	807 (265/542)	1260 (500/760)	Smokers defined as current, past and non-smokers. No further definitions given	Controls were African American adults aged > 35 recruited from ophthalmology clinics at the University of Pennsylvania. Excluded if they had high myopia, family history of POAG, abnormal visual fields, IOP > 21 mmHg, or glaucomatous optic nerve findings. All underwent standardised clinical exams and detailed phenotyping by glaucoma specialists	259 (38.1)	493 (42.4)	Age, race	-	-
Chiam et al., Singapore [33]	Case–control	3499 (1839/1660)	2788 (1361/ 1427)	711 (478/233)	Smokers defined as current, past and non-smokers. No further definitions given	Controls were Chinese Singaporeans aged ≥ 40 from the population-based Singapore Chinese Eye Study (2009–2011). Eligible controls had IOP ≤ 21 mmHg, open angles, healthy optic discs, and normal visual fields. Participants with glaucoma or other ocular diseases were excluded. All underwent comprehensive ophthalmic exams and interviews	359 (12.9)	158 (22.4)	States multiple logistic regression and multivariate analysis	0.83 (0.46 – 1.47) p = 0.520	-
*Cross-sectional Studies*											
Klein et al., USA [39]	Cross-sectional	4926 (N/A)	4822 (N/A)	104 (N/A)	Smoking less than 100 cigarettes in a lifetime was a non-smoker Those who had smoker above 100 and stopped were past smokers Current smokers were participants that currently smoked but no further definitions given	Controls were selected from a population-based cohort of adults aged 43–84 years in Beaver Dam, Wisconsin. Participants underwent standardized ophthalmic assessments including IOP measurement, optic disc photography, and visual field testing. Controls were individuals not meeting criteria for definite or probable POAG based on established diagnostic protocols	-	-	Does not specify	Men 1.18 (0.58 – 2.40) no p-value Women 0.77 (0.39 – 1.53) no p-value	-
Quigley et al., USA [42]	Cross-sectional	4768 (1849/2919)	4674 (1618/ 2858)	94 (33/61)	Smokers were categorised as current, past and non-smokers but no further definitions were given	Controls were Hispanic adults aged ≥ 40 years from randomly selected census block groups in Arizona. Participants completed home interviews and standardized clinic examinations, including IOP, optic disc, and visual field assessments. Controls were those not meeting international diagnostic criteria for POAG	1282 (27.5)	31 (33)	Age and ethnicity	0.96 (0.61 – 1.51) no p-value	-
Khalili et al., Iran [38]	Cross-sectional	11,208 (4992/6216)	11,092 (4934/ 6158)	116 (58/58)	Non-smokers smoked < 100 cigarettes in lifetime, ex-smokers had smoked > 100 and stopped 1 year before study questionnaire, regular smokers smoked at least 1 cigarette a day and heavy smokers smoked > 20 cigarettes a day. In this study regular and heavy smokers are current smokers	Controls were adults aged 35–70 years from the Azar cohort (Iran), who completed comprehensive eye exams and did not meet diagnostic criteria for POAG. Selection was population-based. All underwent optometrist-led screening, with referrals for full ophthalmological assessment based on predefined clinical flags, including IOP > 21 mmHg and family history	915 (8.3)	6 (5.2)	Age	0.51 (0.21 – 1.23) p = 0.135	-

**Table 3:** Baseline characteristics and effect estimates of all included studies that reported current and past smoking combined

Study ID (Author, location and year of publication)	Type of study	Total Patients			Definition of smokers	Control Group Selection	Control Group	POAG Group	Adjustments & variables controlled for in study	Current and Past Smoking Odds Ratio (95% CI)	Current and Past Smoking Relative risk or Risk Ratio unless specified (95% CI)
		Total Participants (M/F)	Control (M/F)	POAG (M/F)			Current and past smokers combined (%)	Current and past smokers combined (%)			
*Cohort Studies*											
Marshal et al., UK, Canada, Australia [52]	Cohort Study	PROGRESSA 355 (158/197) UK Biobank 56,572 (26,024/30548) CLSA 23,226 (11,497/11729)	-	PROGRESSA = 140 UK Biobank = 867 CLSA = N/A	Does not specify accurately. Smoking assumed to be current and past smoking	Controls comprised treatment-naive glaucoma suspects from the PROGRESSA study: adults with open angles and optic disc changes suggestive of glaucoma but normal baseline visual fields. Enrolled between 2012–2021, they had no prior IOP-lowering therapy and underwent standardized six-monthly assessments over five years to monitor treatment initiation	-	-	PROGESSA = age, gender and maximum IOP UK Biobank = age, gender and highest IOP CLSA = age, gender and IOP	0.81 (0.68 – 0.96) p = 0.014	-
Mahmoudinezhad et al., USA [56]	Cohort Study	610 (255/355)	414 (168/246)	196 (87/109)	Smokers were defined as ‘ever reported smoking’ which was current smoking and past smoking	Controls comprised eyes from the DIGS and ADAGES cohorts: adults (> 18 years) with open angles and suspected glaucoma—defined by elevated IOP or optic nerve appearance—but normal baseline visual fields. Eligible eyes had ≥ 3 reliable VF tests and ≥ 1 year follow-up and remained glaucoma-free throughout monitoring	158 (38.2)	91 (46.4)	Mean IOP, current alcohol consumption, BMI	-	Hazard Ratio 1.15 (0.80 – 1.65) P = 0.439
*Case–control Studies*											
Kaimbo, Buntinx & Missotten, Congo [50]	Case–control	144 (97/47)	104 (67/37)	40 (30/10)	Smoking defined as current smoker or use within 5 years	Controls were selected via systematic sampling of every 10th consecutive patient aged ≥ 30 at Kinshasa ophthalmology clinics, presenting with non-glaucomatous eye conditions. They had normal visual fields and IOP < 21 mmHg, underwent slit-lamp examination, tonometry, and Goldmann perimetry under standard diagnostic protocols, and remained glaucoma-free	13 (12)	8 (20)	Age and BMI	1.9 (0.61 – 5.70) p = 0.22	-
Fan et al., China [49]	Case–control	128 (52/76)	96 (39/57)	32 (13/19)	Smokers were defined as those who smoked more than 5 cigarettes a day for more than 1 year	Controls were frequency-matched clinic attendees without POAG, aged ≥ 40, drawn contemporaneously from the same hospital and outpatient eye clinics. They underwent identical examination protocols—including slit-lamp biomicroscopy, tonometry, gonioscopy, and Goldmann perimetry—and had normal visual fields and IOP ≤ 21 mm Hg at baseline, remaining glaucoma-free during follow-up	-	-	Family history, alcohol intake, hypertension, myocilin sequence	10.8 (1.85 – 63.0) p = 0.008	-
Nilforooshan et al., Iran [55]	Case–control	191 (99/92)	131 (66/65)	60 (33/27)	Smoking was defined as any current use or use within a five-year period	Controls were age- and sex-matched patients ≥ 30 years, randomly sampled from the same ophthalmology clinics and period. They presented with non-glaucomatous eye conditions (e.g., presbyopia, cataract), underwent identical interviews and examinations—including IOP measurement, gonioscopy, optic disc evaluation, and perimetry—and remained free of POAG	13 (9)	10 (16)	-	1.81 (0.74 – 4.41) p = 0.184	-
Graham et al., Australia [54]	Case–control	**All glaucoma + controls 176 (95/81) POAG + controls 140 (N/A)	50 (26/24)	90 (55/35)	Does not specify accurately. Smoking history assumed to be current and past smoking	Controls comprised 66 healthy adults (> 18 years) recruited from the same university clinic and laboratory. They were age- and sex-matched, had no ocular disease or family history of glaucoma, and exhibited normal IOP, optic nerve appearance, and visual fields. All underwent identical assessments including gonioscopy, tonometry, blood pressure, and retinal imaging	11 (22)	40 (44)	Age, gender and BMI	-	Regression coefficient 0.226, standard error 0.081 p = 0.006
*Cross-sectional Studies*											
Zhou et al., USA [53]	Cross-sectional	405 (204/201)	113 (59/54)	292 (145/147)	Smoking was defined as smoking history which was current smoking and past smoking	???	53 (46.9)	86 (29.5)	-	0.36 (0.17 – 0.78) p = 0.0095	-
Liu et al., China (2022)	Cross-sectional	48,398 (20,456/ 27,942)	-	279 (N/A)	Does not specify accurately. Smoking assumed to be current and past smoking	Controls comprised rural Chinese residents aged ≥ 6 years who screened negative for glaucoma. All underwent visual acuity testing, slit-lamp examination, ophthalmoscopy, and non-contact tonometry; those without suspicious findings served as controls, while suspects received fundus photography, Goldmann tonometry, perimetry, and gonioscopy to confirm absence of disease. Analysis was divided into all residents > 6 and residents > 40 years old (included in this analysis)	-	-	Age, education, gender, income, marital status, occupation and disease history	1.458 (0.979 – 2.173) no p-value	-

**Table 4:** Baseline characteristics and effect estimates of all included studies that reported smoking doses

Study ID (Author, location and year of publication)	Type of study	Total Patients			Definition of smokers		Control Group	POAG Group	Adjustments & variables controlled for in study	Smoking dose Odds ratio (95% CI)	Smoking dose Relative risk or risk ratio unless specified (95% CI)
		Total Participants (M/F)	Control (M/F)	POAG (M/F)		Control Group Selection	Smoking dose	Smoking dose			
*Cohort Studies*											
Kang et al., USA [36]	Prospective cohort	71,819 (28,139/43680)	71,369 (N/A)	450 (N/A)	Smokers defined as current, past and non-smokers. No further definitions given	Controls were drawn from two large US cohorts (NHS and HPFS) of health professionals aged ≥ 40 years, free of glaucoma at baseline, and who reported undergoing eye examinations during follow-up. Eligibility was updated biennially. The cohorts were predominantly White, and data were collected through validated, repeated questionnaires	-	Never smoked = 221(49.1) 1–9 pack years = 100 (22.2) 10 – 19 pack years = 55 (12.2) 20 – 29 pack years = 33 (7.6) > 30 pack years = 40 (8.9)	Age, sex, BMI, hypertension, diabetes, ethnicity and alcohol intake	-	1–9 pack years 1.12 (0.66–1.92) 10–19 pack years 0.72 (0.53—0.98) 20–29 pack years 0.85 (0.59—1.23) > 30 pack years 0.78 (0.55—1.11) p-value for linear trends = 0.06
Doshi et al., USA [34]	Retrospective cohort	6142 (N/A)	5624 (N/A)	289 (N/A)	Smokers defined as current, past and non-smokers. No further definitions given	Controls were self-identified Latino participants aged ≥ 40 from six census tracts in La Puente, California, who completed both home interviews and in-clinic examinations and were found to have neither OAG nor OHT	0–1 Pack years = 3568 (63.4) 1–9 Pack years = 1158 (20.6) 10–19 pack years = 301 (5.4) 20–29 pack years = 179 (3.2) > 30 = 239 (4.2)	0–1 Pack years = 184 (63.7) 1–9 Pack years = 51 (17.6) 10–19 pack years = 17 (5.9) 20–29 pack years = 6 (2.1) > 30 = 25 (8.7)	Age and gender	1–9 Pack years 0.85 (0.59—1.21) no p-value 10–19 pack years 1.05 (0.59—1.86) no p-value 20–29 pack years 0.47 (0.2—1.14) no p-value > 30 pack years 1.02 (0.62—1.67) no p-value	-
Wise et al., USA [48]	Prospective cohort	32,570 (0/32,570)	32,204 (0/32204)	366 (0/366)	Smokers defined as current, past and non-smokers. No further definitions given	Controls were African-American women aged 21–69 from the Black Women’s Health Study, who completed follow-up questionnaires and did not develop POAG. Only those reporting recent eye exams were included to minimise detection bias. Selection was prospective, updated biennially, and based on physician-confirmed non-cases within the analytic cohort	-	Never = 201 < 10 pack years = 65 (17.8) 10–14 pack years = 18 (4.9) 15–19 pack years = 22 (6.0) > 20 pack years = 55 (15.0)	Alcohol consumption, diabetes, BMI, age, ex, weight, height and education	-	Incidence rate ratio < 10 pack years 0.94 (0.71—1.25) 10–14 pack years 0.72 (0.44—1.16) 15–19 pack years 0.93 (0.59—1.46) > 20 pack years 1.08 (0.79—1.47) p = value for ordinal trend = 0.32
Perez-de-Arcelus et al., Spain [41]	Prospective cohort	16,797 (6683/10114)	16,613 (N/A)	184 (N/A)	Smokers defined as current, past and non-smokers. No further definitions given	Controls were university graduates from the SUN cohort who were free of glaucoma at baseline and had complete smoking data. Follow-up was via biennial questionnaires. Those with glaucoma at baseline or implausible energy intakes were excluded. Cohort included mainly Caucasian, highly educated individuals, with 50% being health professionals	-	0.1—2.5 Pack years = 8 1.6—6 pack years = 11 6.1—11.5 pack years = 16 11.5—20.5 pack years = 25 20.6—102.5 pack years = 43	Age, sex, BMI, hypertension, T2DM, physical activity, coffee consumption, alcohol, Mediterranean diet, total energy intake	-	Hazard ratio 0.1—2.5 Pack years 0.83 (0.39—1.75) 2.6—6 pack years 1.28 (0.67—2.46) 6.1—11.5 pack years HR 1.55 (0.88–2.73) 11.5—20.5 pack years HR 1.57 (0.96—2.57) 20.6—102.5 pack years HR 1.70 (1.10—2.64) p-value for trend = 0.009

### Smoking definitions

The definition of smoking showed heterogeneity between studies. Some authors defined smokers as either current or past smokers, whereas others were ambiguous in their definition, or defined a ‘smoker’ as someone who currently or previously smoked but did not differentiate between them. Eighteen studies [31–48] involved current smokers; twelve [31–36, 41–44, 47, 48] of these did not provide any additional definitions, while six studies offered their own definitions of a current smoker [37–40, 45, 46]. These definitions were, ‘current smoker was an individual who currently smoked > 20 cigarettes a day,’ [40] ‘current smokers either smoked 20 cigarettes a day for 10 or more years, or 10–20 cigarettes a day for 15 or more years,’ [37] ‘current smokers were participants that currently smoked but smoking less than 100 cigarettes in a lifetime was a non-smoker,’ [39] ‘active smokers were defined as smoking five or more cigarettes a day for the past year or more,’ [46] ‘those who smoked daily for a minimum of 60 days before the enrolment of the study,’ [45] and, ‘regular smokers smoked at least 1 cigarette a day and heavy smokers smoked > 20 cigarettes a day.’ [38] (Table 1).

Eleven studies [31–34, 36, 38, 39, 41–43, 48] included past smokers, nine of which did not give any further definitions of a past smoker [31–34, 36, 41–43, 48]. One study defined a past smoker as, ‘a smoker who had smoked above 100 cigarettes and stopped,’ [39] and another defined a past smoker as smoker who had smoked > 100 and stopped 1 year before the study questionnaire [38] (Table 2).

Eight studies [49–56] grouped current and past smokers together (Table 3). Five of these studies gave clear definitions, which were, ‘smoking defined as current smoker or use within 5 years,’ [50] ‘smokers defined as those who smoked more than five cigarettes a day for more than one year,’ [49] ‘smoking defined as any current use or use within a five-year period,’ [55] ‘smoking defined as smoking history which was current and past smoking’ [53] and ‘smoking defined as ever reported smoking’ [56]. The other three studies did not give clear definitions of what smoking was and therefore they were assumed to include current and past smoking [51, 52, 54]. These inconsistencies likely contribute to exposure misclassification and reduce comparability between studies. Regarding the study by Fan et al. [49], from the definition of smoking it was difficult to ascertain if they were referring to current and past smokers combined or current smokers only. However, on reviewing the literature, a previous systematic review published in 2017 [25] had categorised their definition into current and past smokers combined, and thus this study was categorised into ‘current and past smokers combined’ cohort. (Table 3).

## Meta-analysis

### Current smokers vs non-smokers

**Fig. 2:**
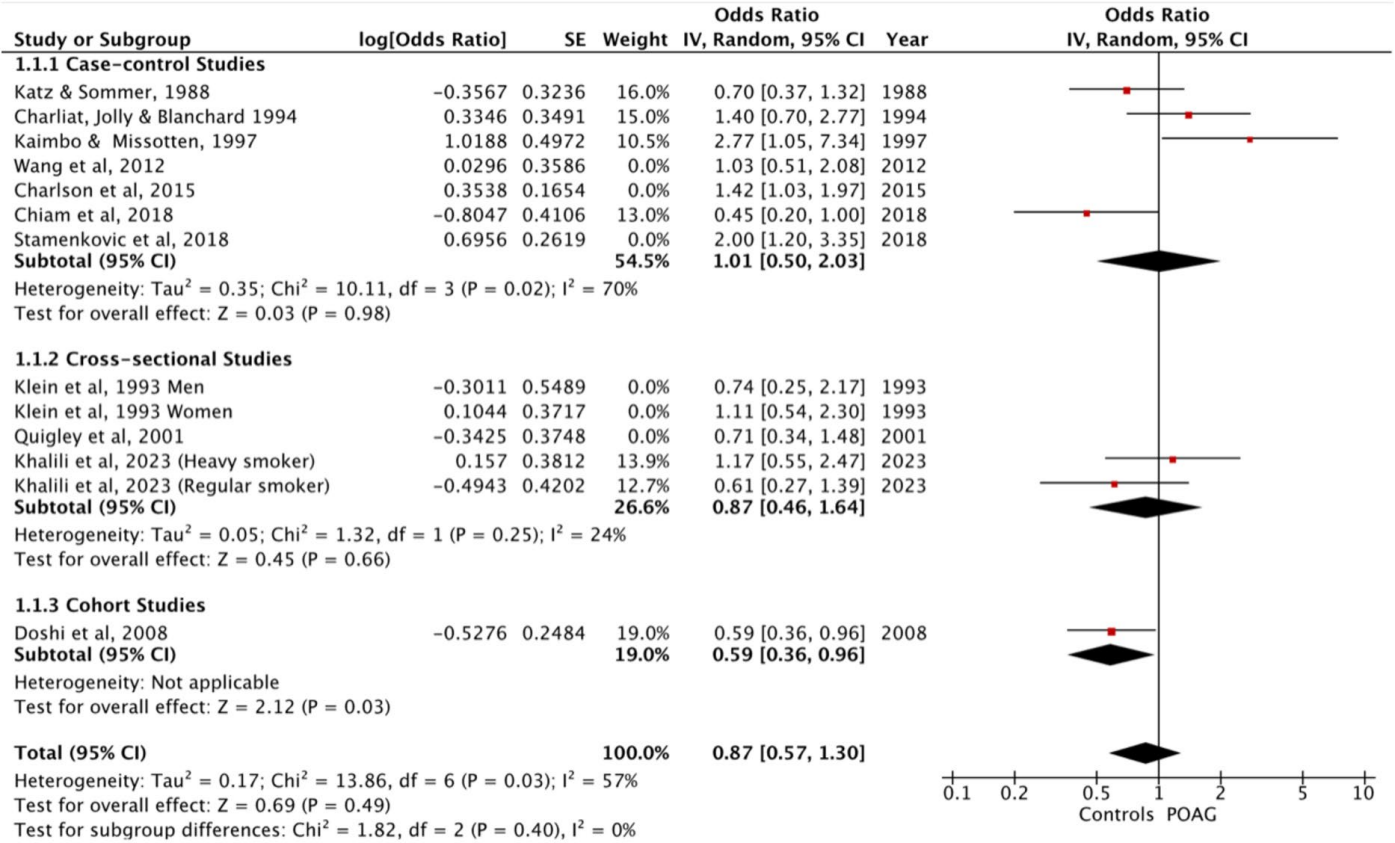
Meta-analysis of current smokers vs non-smokers with sub-group analysis based on type of study. Sensitivity analyses of current smokers vs non-smokers excluding studies at high overall risk of bias

Eleven studies, comparing current smokers with non-smokers reported OR [31–35, 37–39, 42, 45, 46]. The meta-analysis showed that current tobacco smoking was not significantly associated with POAG (OR = 1.00, 95%CI 0.76 – 1.33, *p* = 0.97), (current smokers in POAG group = 430 / 2963 [excludes Kaimbo [35] and Klein [39] from n numbers as n numbers not reported], total participants in studies = 33,960) (Fig. 2). There was moderate heterogeneity between studies (*p* = 0.005) as indicated by an I^2^ value of 58%. Pooling ‘any smoking history’ necessarily aggregates heterogeneous exposure constructs (current, former, and mixed/ambiguous definitions), which may contribute to the observed between-study heterogeneity. Sub-group analysis based on type of study demonstrated that case–control studies and cross-sectional studies also showed that current tobacco smoking was not significantly associated with POAG (OR = 1.21, 95%CI 0.83—1.77, *p* = 0.33 and OR = 0.86, 95%CI 0.61 – 1.23, *p* = 0.42 respectively). One cohort study [34] exhibited an OR of 0.59, 95%CI – 0.96, *p* = 0.03. Three studies [36, 40, 44] reported RRs, but two of these did not state 95%CIs [40, 44].

Four studies reported different effect values [41, 43, 47, 48]. Wilson et al. [47] reported a rate ratio of 2.9 (95%CI 1.3–6.6), Wise et al. [48] reported an incidence rate ratio (IRR) of 0.97 (95%CI 0.7 – 1.35), Perez-de-Arcelus, [41] reported a hazard ratio (HR) of 1.88 (95%CI 1.26 – 2.81, *p* = 0.002) and Ramdas et al. [43] reported a Beta value of − 0.40 (0.49) in males (*p* = 0.41) and − 0.22 (0.21) in females (*p* = 0.29) (Table 1).

In sensitivity analyses excluding studies at high overall risk of bias, the findings remained non-significant (OR = 0.87, 95%CI 0.57—1.30, *P* = 0.49) (Fig. 2).

### Past smokers vs non-smokers

**Fig. 3:**
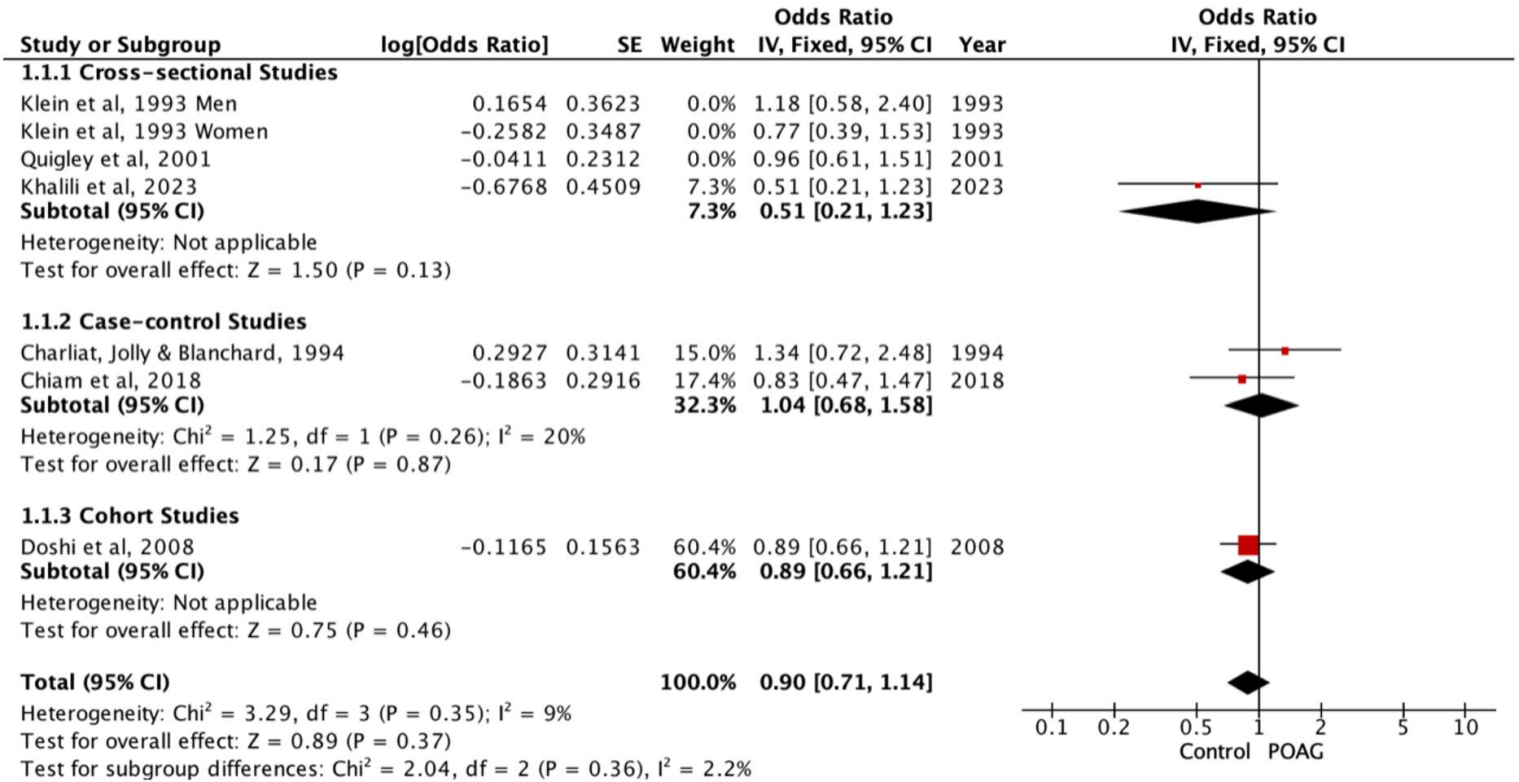
Meta-analysis of past smokers vs non-smokers with sub-group analysis based on type of study. Sensitivity analyses of past smokers vs non-smokers excluding studies at high overall risk of bias

Past smoking vs non-smoking was compared by six studies which reported ORs [31, 33, 34, 38, 39, 42]. The meta-analysis showed that there was no significant association between past smoking and POAG (OR = 0.92, 95%CI 0.75 – 1.11, *p* = 0.38), (past smokers in POAG = 333 / 1385 [excludes Klein [39] from n numbers as n numbers not reported], total participants in studies = 30,893) (Fig. 3), and there was low heterogeneity between these studies (*p* = 0.66, I^2^ = 0%). Sub-group analysis based on type of study demonstrated that case–control studies and cross-sectional studies also showed that past smoking was not significantly associated with POAG (OR = 1.04, 95%CI 0.68 – 1.58, *p* = 0.26 and OR = 0.88, 95%CI 0.65 – 1.20, *p* = 0.43 respectively). One cohort study exhibited an OR of 0.89, 95%CI 0.66 – 1.21, *p* = 0.46).

Only one study reported RRs for past smoking vs non-smoking: RR 0.91, 95%CI 0.63 – 1.32 (past smokers in POAG = 181 / 450, total participants = 71,819) [36].

Three studies reported different effect values [41, 43, 48]. Wise et al. [48] reported an IRR of 0.89 (95%CI 0.70 – 1.13), Perez-de-Arcelus, [41] reported a HR of 1.27 (95%CI 0.88 – 1.82, *p* = 0.198) and Ramdas et al. [43] reported a Beta value of − 0.82 (0.38) in males (*p* = 0.03) and 0.06 (0.16) in females (*p* = 0.70).

In sensitivity analyses excluding studies at high overall risk of bias, the findings remained non-significant (OR = 0.90, 95%CI 0.71–1.14, *P* = 0.37) (Fig. 3).

### Current and past smokers combined vs Non-smokers

**Fig. 4:**
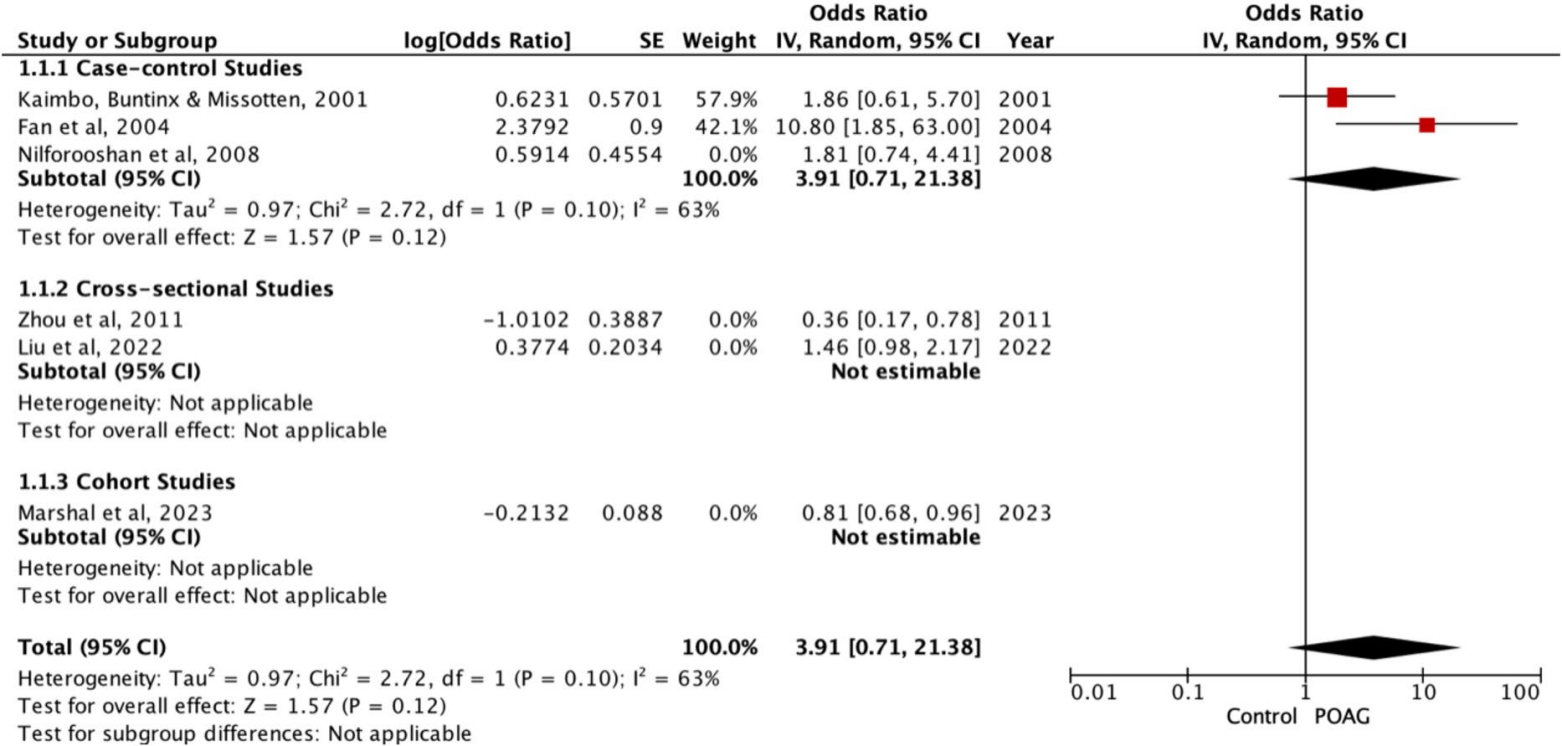
Meta-analysis of current and past smoking combined vs non-smokers with sub-group analysis based on type of study. Sensitivity analyses of current and past smoking combined vs non-smokers excluding studies at high overall risk of bias

OR for current and past smokers combined vs non-smokers was reported in six studies [49–53, 55]. Between these studies, there was high heterogeneity (*p* < 0.0002, I^2^ = 79%), and there was no significant association between current and past smoking combined and POAG (OR = 1.21, 95%CI 0.70 – 2.07, *p* = 0.49), (combined smokers in POAG = 104 / 392 [excludes Fan [49], Liu 2022 and Marshal [52] from n numbers as no n numbers reported], total participants in studies = 129, 419) (Fig. 4). This heterogeneity is plausibly driven in part by non-comparable exposure definitions across studies (e.g., ‘ever’ smoking vs ‘current or recent’ smoking), including studies that did not provide clear categorisation criteria. Sub-group analysis based on type of study demonstrated that cross-sectional studies showed that there was no statistically significant association with POAG (OR = 0.76, 95%CI 0.20 – 2.95, *p* = 0.69, but there was for case–control studies (OR = 2.59, 95%CI 1.06 – 6.31, *p* = 0.04). However, this was based on only 2 studies with a moderate level of heterogeneity. One cohort study exhibited an OR of 0.81, 95%CI 0.70 – 0.96, *p* = 0.49).

There was one study that reported a regression coefficient of 0.226 (standard error 0.081, *p* = 0.006) [54] and one study that reported a HR of 1.15 (95%CI 0.80–1.65, *p* = 0.439) [56] (Table 3).

In sensitivity analyses excluding studies at high overall risk of bias, the findings remained non-significant (OR = 3.91, 95%CI 0.71–21.38, *P* = 0.12) (Fig. 4).

### Any smoking history vs non-smokers

**Fig. 5:**
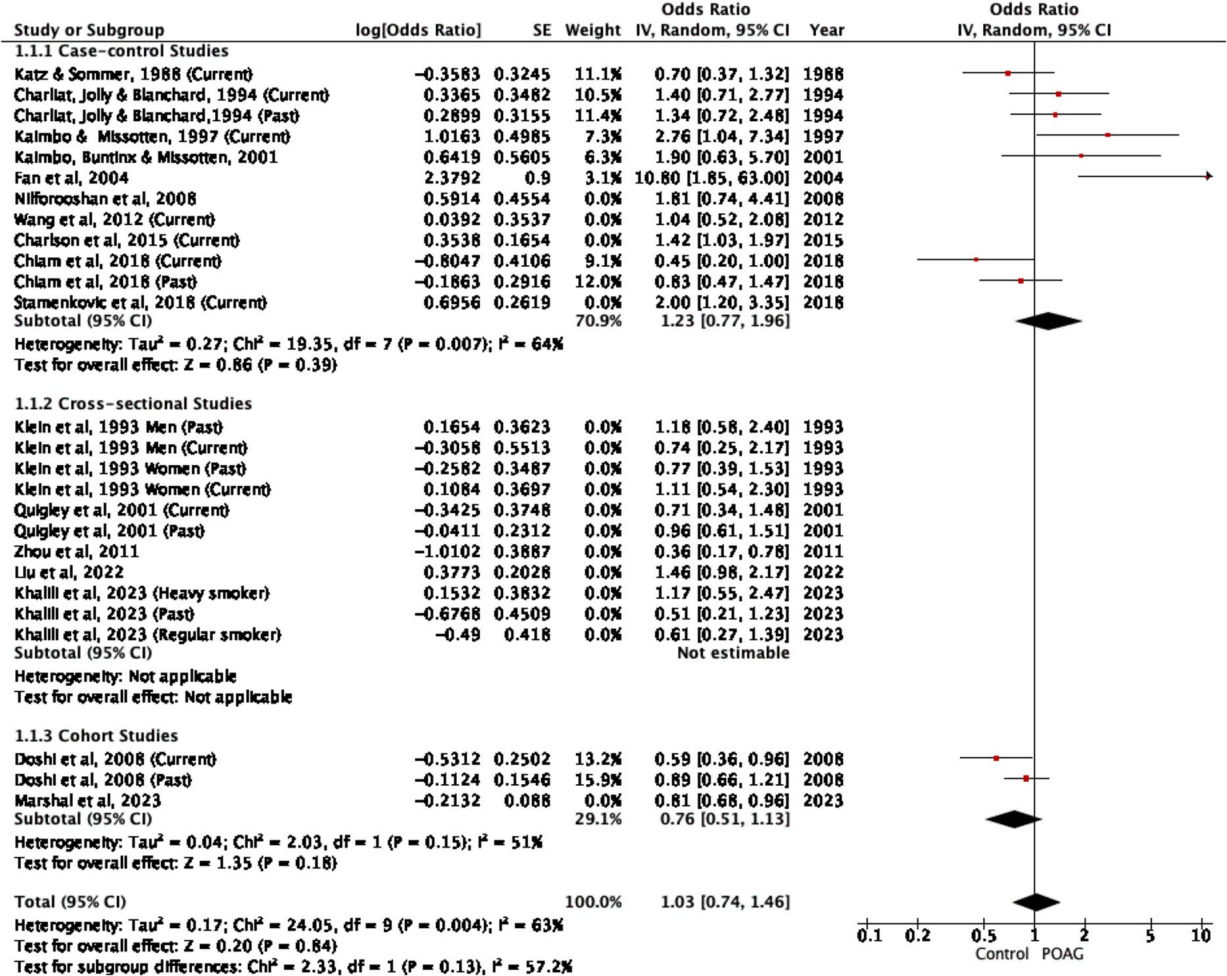
Meta-analysis of any smoking history vs non-smokers with sub-group analysis based on type of study. Sensitivity analyses of any smoking history vs non-smokers excluding studies at high overall risk of bias

To determine if any smoking history was associated with POAG, studies that reported ‘current smokers’, ‘past smokers’ and ‘current and past smokers combined’, were pooled together for meta-analysis. A total of 17 studies [31–35, 37–39, 45, 46, 49–52, 52, 53, 55] were included with 26 ORs for which there was high heterogeneity (*p* = 0.0001, I^2^ = 58%). The overall effect estimate showed no significant association between any smoking history and POAG (OR = 1.00, 95%CI 0.84 – 1.19, *p* = 1.00) (any smoking history in POAG = 867 / 3355 [excludes Kaimbo [35], Klein [39], Fan [49], Liu 2022 and Marshal [52] from n numbers as no n numbers reported], total participants in studies = 163, 379) (Fig. 5). Sub-group analysis based on type of study demonstrated that case–control studies and cross-sectional studies also showed that past smoking was not significantly associated with POAG (OR = 1.30, 95%CI 0.97 – 1.76, *p* = 0.08 and OR = 0.87, 95%CI 0.68 – 1.13, *p* = 0.31 respectively). Two cohort studies (3 ORs in total) exhibited an overall OR of 0.80, 95%CI 0.69 – 0.93, *p* = 0.003).

In sensitivity analyses excluding studies at high overall risk of bias, the findings remained non-significant (OR = 1.03, 95%CI 0.74- 1.46, *P* = 0.84) (Fig. 5).

### Smoking dose and POAG

Across four studies reporting pack-year exposure, the pooled estimate did not demonstrate a statistically significant association with POAG; however, between-study heterogeneity was extreme and effect directions varied, limiting interpretability [34, 36, 41, 48]. One study reported OR of 0.85 (95%CI 0.59 – 1.21) for 1–9 pack years, 1.05 (95%CI 0.59 – 1.86) for 10–19 pack years, 0.47 (95%CI 0.2 – 1.14) for 20–29 pack years and 1.02 (95%CI 0.62 – 1.67) for > 30 pack years (Table 4).

Kang et al. [36] reported RRs of 1.12 (95%CI 0.66 – 1.92) for 1–9 pack years, 0.72 (95%CI 0.53 – 0.98) for 10 – 19 pack years, 0.85 (95%CI 0.59 – 1.23) for 20 – 29 pack years and 0.78 (95%CI 0.55 – 1.11) for > 30 pack years (p-value for linear trend = 0.06) (Table 4).

Wise et al. [48] reported IRRs. For < 10 pack years the IRR was 0.94 (95%CI 0.71 – 1.25), for 10–14 pack years it was 0.72 (95%CI 0.44 – 1.16), for 15–19 pack years it was 0.93 (95%CI 0.59 – 1.46) and for > 20 pack years it was 1.08 (95%CI 0.79 – 1.47) (Table 4).

The fourth study reported HRs [41]. For 0.1 – 2.5 pack years the HR was 0.83 (95%CI 0.39 – 1.75), for 2.6 – 6 pack years 1.28 (95%CI 0.67 – 2.46), for 6.1 – 11.5 pack years 1.55 (95%CI 0.88 – 2.73), for 11.5 – 20.5 pack years 1.57 (95%CI 0.96 – 2.57) and for 20.6 – 102.5 pack years 1.70 (95%CI 1.10 – 2.64) (*p*-value for trend = 0.009) (Table 4).

### Risk of bias and quality of evidence

The risk of bias was deemed to be ‘high risk’ in 14 studies [32, 36, 38, 39, 42, 44–46, 48, 51–55] and ‘some concerns’ in 12 studies [31, 33–35, 37, 40, 41, 43, 47, 49, 50, 56] (Table 5). The main domain that did not contribute to a higher risk of bias was post exposure intervention, while all the other domains did. The quality of evidence, assessed by GRADE analysis, was rated as moderate for past smokers; low for current smokers and any smoking history; and very low for current and past smokers combined. (Table 6).

**Table 5:** Risk of bias for all included studies using the ROBINS-E tool

Study ID (Author, location and year of publication)	Domain 1 Confounding	Domain 2 Exposure	Domain 3 Selection of participants	Domain 4 Post exposure interventions	Domain 5 Missing data	Domain 6 Outcome	Domain 7 Reported result	Overall Risk of Bias
Morgan & Drance, Canada [40]	Some concerns	Low risk	Some concerns	Low risk	Low risk	Low risk	Some concerns	Some concerns
Reynolds, USA [44]	Some concerns	Some concerns	Some concerns	Low risk	Low risk	Some concerns	Low risk	High risk
Wilson et al., USA [47]	Low risk	Low risk	Some concerns	Low risk	Some concerns	Low risk	Low risk	Some concerns
Katz & Sommer, USA [37]	Some concerns	Low risk	-Some concerns	Low risk	Low risk	Low risk	Some concerns	Some concerns
Klein et al., USA [39]	High risk	Some concerns	Some concerns	Low risk	High risk	Some concerns	High risk	High risk
Charliat, Jolly & Blanchard, France [31]	Low risk	Some concerns	Some concerns	Low risk	Low risk	Low risk	Some concerns	Some concerns
Kaimbo & Missotten, Congo [35]	Low risk	Low risk	Some concerns	Low risk	Low risk	Some concerns	Some concerns	Some concerns
Quigley et al., USA [42]	High risk	Low risk	Some concerns	Low risk	Low risk	Low risk	Some concerns	High risk
Kaimbo, Buntinx & Missotten, Congo [50]	Low risk	Some concerns	Some concerns	Low risk	Low risk	Some concerns	Low risk	Some concerns
Kang et al., USA [36]	High risk	Low risk	High risk	Low risk	Some concerns	Low risk	Low risk	High risk
Fan et al., China [49]	Some concerns	Some concerns	Some concerns	Low risk	Low risk	Low risk	Low risk	Some concerns
Doshi et al., USA [34]	Low risk	Low risk	Some concerns	Low risk	Low risk	Low risk	Low risk	Some concerns
Nilforooshan et al., Iran [55]	High risk	Some concerns	High risk	Low risk	Some concerns	Some concerns	Low risk	High risk
Wise et al., USA [48]	High risk	-Some concerns	Some concerns	Some concerns	High risk	Low risk	Some concerns	High risk
Ramdas et al.,								
Netherlands [43]	Low risk	Some concerns	Some concerns	Low risk	Low risk	Low risk	Low risk	Some concerns
Zhou et al., USA [53]	Low risk	Some concerns	High risk	Some concerns	High risk	Low risk	Low risk	High risk
Wang et al., China [46]	High risk	Some concerns	Some concerns	Low risk	Low risk	Some concerns	Low risk	High risk
Graham et al., Australia [54]	Some concerns	Some concerns	High risk	Some concerns	Low risk	High risk	Some concerns	High risk
Charlson et al., USA [32]	Some concerns	Some concerns	High risk	Low risk	Some concerns	High risk	High risk	High risk
Perez-de-Arcelus et al., Spain [41]	Low risk	Low risk	Some concerns	Low risk	Some concerns	Some concerns	Low risk	Some concerns
Stamenkovic et al., Serbia [45]	Some concerns	Some concerns	High risk	Low risk	Some concerns	Some concerns	Some concerns	High risk
Chiam et al., Singapore [33]	Low risk	Low risk	Some concerns	Low risk	Some concerns	Low risk	Low risk	Some concerns
Liu et al., China (2022)	Low risk	Some concerns	High risk	Low risk	Low risk	Some concerns	Some concerns	High risk
Marshal et al., UK, Canada, Australia [52]	Some concerns	Some concerns	High risk	Low risk	Some concerns	Some concerns	Some concerns	High risk
Khalili et al., Iran [38]	Some concerns	Some concerns	High risk	Low risk	Low risk	Some concerns	High risk	High risk
Mahmoudinezhad et al., USA [56]	Some concerns	Low risk	Some concerns	Low risk	Low risk	Low risk	Some concerns	Some concerns

**Table 6:** Quality of evidence of each outcome as assessed by the GRADE system

Outcomes	No. of studies	Risk of bias	Imprecision	Inconsistency	Indirectness	Publication Bias	Overall GRADE Rating
Current smokers vs non-smokers	11	Very High	Moderate	High	Low	Low	Low
Past smokers vs non-smokers	6	High	Moderate	Low	Low	Low	Moderate
Current and past smoking combined	6	Very High	Moderate	Very High	Low	Low	Very Low
Any smoking history	17	Very High	Moderate	High	Low	Low	Low

## Discussion

### Summary of results

Of the 26 studies included in this systematic review and meta-analysis, seven [33, 38, 41, 45, 51, 52, 56] were published since the last systematic review in 2017 [25]. Our meta-analyses revealed that current smokers, former smokers, and those with both current and past smoking histories showed no statistically significant association with the prevalence of POAG when compared to individuals who had never smoked. However, a high risk of bias was present in 14 of the 26 studies [34, 36, 38, 39, 42, 44–46, 48, 51–55]. Notably, one population-based cohort study [33] reported a statistically significant inverse association for current smoking, underscoring heterogeneity despite the null pooled estimate.

From the four studies that evaluated the association between pack years and POAG, three showed no association [24, 26, 48]. Only one study identified a relationship between smoking from 20.6 to 102.5 pack years and an increased risk of POAG, with a HR of 1.70 (95%CI 1.10 – 2.64) [41]. Evidence on dose–response (pack-year) relationships is currently insufficient, with very high heterogeneity, suggesting meaningful differences in exposure ascertainment, categorisation, and confounding control. Accordingly, no robust conclusions about a pack-year dose–response relationship can be drawn from the available literature.

### Previous systematic reviews

The literature published to date remains discordant, with conflicting results. A meta-analysis in 2004 by Bonovas et al. [17] concluded that current smoking significantly increased the risk of POAG, with an OR of 1.37 (95%CI 1.00 – 1.87), while past smoking did not (OR = 1.03, 95%CI 0.77 – 1.38) [17]. However, this review only included six studies, therefore an addition of nearly 20-years of new information within the field, likely explained the differing results we have identified. Furthermore, unadjusted RRs were also employed by Bonovas et al. [17]; inherently, these may introduce bias and have the potential to skew results.

Following this meta-analysis, a systematic review was published in 2008 by Edwards et al. [18]. This review included 11 studies, but the authors did not carry out a meta-analysis. Instead, they conducted a narrative synthesis of the results of the individual papers and concluded that there was ‘no evidence of a strong or consistent association between smoking and POAG’ [18] which agrees with our meta-analysis findings.

The next systematic review to be carried out was in 2016 by Zhou et al. [19]. The authors did conduct a meta-analysis but had strict inclusion criteria. In particular, they only included studies that included and classified individuals as current smokers, past smokers and never smokers. Furthermore, they only included studies that reported ORs or RRs. As a result, the authors included only six studies, with the results suggesting that current smokers ‘were not significantly associated with the risk of POAG (pooled RR = 0.97, 95%CI 0.81 – 1.16, *p* = 0.75),’ similarly, past smoking was not associated with POAG (RR = 0.97, 95%CI 0.83 – 1.13, *p* = 0.66). These findings are also consistent with our meta-analyses.

A recent systematic review was published by Jain et al. [25] and included 17 studies up to February 2015. Of these, only three studies indicated a strong association between current smoking and POAG [35, 37, 47] while some showed an association between past smoking and POAG [57, 58]. These two studies were excluded from our review because one used patients with ocular hypertension (OHT) as controls [58] and the other categorised patients into ‘at least probable’ and ‘at least possible’ glaucoma, meaning that not all patients in the glaucoma groups had a confirmed diagnosis of POAG [57]. Jain et al. [25] concluded that there was ‘very limited evidence for a causal association between tobacco smoking and POAG’ [25], which again mirrors the results of our systematic review and meta-analysis.

### Risk factors for POAG

Currently established risk factors for POAG include age, African-Caribbean descent, myopia, family history and IOP [2, 5–11]. More recently, genetic work has identified numerous open-angle glaucoma loci with consistent effects across ancestries (GLC1) [59]. IOP reduction remains the only proven modifiable factor for slowing POAG progression; whether modifying other systemic factors reduces incident disease is not established [60]. Other vascular risk factors that have been explored include systemic blood pressure and diabetes mellitus (DM) [61]. Findings for blood pressure are contrasting: several studies report that systemic hypertension (elevated systolic and/or diastolic blood pressure) is associated with higher POAG risk [62, 63], whereas low blood pressure—particularly nocturnal hypotension and reduced ocular perfusion pressure—has also been linked to POAG development and/or faster progression [61]. A plausible interpretation is a U-shaped relationship, where both extremes of blood pressure may be detrimental through vascular dysregulation of the optic nerve head. In DM, long-standing hyperglycaemia can dysregulate blood flow to retinal vascular endothelial cells [64] and may exacerbate connective-tissue remodelling at the trabecular meshwork, increasing IOP [65]. While earlier meta-analyses suggest higher POAG risk among people with diabetes [66, 67], other reviews report differing results [61].

The hypothesised mechanisms linking cigarette smoking to POAG resemble those proposed for hypertension and DM: vasoconstriction of episcleral veins with increased outflow resistance and IOP [68]; alteration of vasoactive mediators (e.g., endothelin-1, neuropeptide Y, nitric oxide, prostacyclin) with downstream effects on blood pressure and ocular perfusion [69]; and oxidative injury to the trabecular meshwork and retinal ganglion cells [70]. Despite these biologically plausible pathways, our meta-analysis found no association between smoking and POAG. Had an association been present, smoking would represent a modifiable target for reducing incident POAG, analogous to IOP lowering for progression. This lack of association with smoking does not negate the role of vascular dysregulation in POAG; rather, it suggests that smoking itself is not a risk factor for the disease.

Therefore, It is important to consider why smoking may not be linked with an increased relative risk of developing POAG in our comprehensive study. One possible explanation is that nicotine primarily affects intraocular pressure transiently. However, the current literature lacks conclusive evidence regarding the permanence of IOP changes induced by nicotine. The studies primarily focus on short-term effects and do not explore whether these changes persist over time or result in chronic conditions such as POAG. For instance, the Blue Mountains Eye Study [71] revealed that current smokers had slightly higher mean IOP (16.34 mmHg) compared to non-smokers (16.04 mmHg) after adjusting for various factors. Although cardinal suggest that the risk of progression decreases by 10% with each 1 mmHg IOP reduction, the impact of the small difference in IOP between smokers and non-smokers, may not be detectible by studies. In contrast, it may well be that the 0.3 mmHg difference is too insignificant to substantially impact the risk of developing POAG [71].

POAG is believed to develop as a consequence of elevated IOP or vascular issues that cause ongoing stress and damage to retinal ganglion cells [72]. The auto-regulatory mechanism of the optic nerve head maintains stable blood flow despite fluctuations in IOP and diurnal variation. If nicotine were to induce temporary changes in IOP, this buffering process might account for why it does not result in vascular compromise of the optic nerve head. This could mitigate the anticipated harmful effects on optic nerve health and potentially avert an increased risk of POAG. However, the long-term effects of nicotine on optic nerve vascular autoregulation are not explicitly addressed in the literature, and further research is warranted to further understand this aspect.

From a genetic perspective, single nucleotide polymorphisms (SNPs) such as rs1063192 and rs10483727 have been identified, which were found to have protective effects against POAG [73]. Genetic susceptibility and comorbidities may modify how smoking-related vascular and oxidative stressors interact with POAG pathophysiology, potentially contributing to inter-individual variability. This variability and the multifactorial nature of POAG could obscure a connection between smoking and POAG in population-based studies.

More recent genetic studies have evaluated associations between smoking-related traits and glaucoma. Using genetic epidemiology approaches (including Mendelian randomisation and genetic correlation analyses), studies drawing on resources such as the UK Biobank and the Rotterdam Study have reported, at most, weak and inconsistently directed associations between smoking-related traits and glaucoma phenotypes. In analyses using the UK Biobank and the Rotterdam Study [74], results have shown a nuanced relationship, showing a consistently weak inverse correlation between smoking and glaucoma traits, although these findings were not statistically significant after correction. Some analyses have reported signals suggesting lower IOP with genetically predicted smoking initiation or lower POAG risk with smoking intensity; however, these findings are not consistent across traits/outcomes and should be interpreted as contextual rather than definitive evidence. Any apparent “protective” direction of association should be considered hypothesis-generating rather than confirmatory. These results, although surprising, are not the first time this association has been suggested. The randomised placebo-controlled United Kingdom Glaucoma Treatment Study (UKGTS) similarly found an inverse association between smoking initiation and decreased rates of glaucoma progression based on visual field testing [75]. This finding should also be considered hypothesis-generating and does not establish a protective causal effect. Overall, the genetic literature does not provide a clear, consistent causal signal linking smoking liability to POAG, and it does not materially alter the interpretation of our pooled observational estimates.

Studies such as this [74, 75] shed light on the intricate genetic factors influencing both smoking behaviours and glaucoma susceptibility, providing valuable insights into the intersection of genetics, smoking, and eye health. Nonetheless they have their limitations. Issues concerning data accuracy, such as reliance on self-reported smoking information, could introduce bias and measurement errors, potentially affecting the study's outcomes. Furthermore, there is the possibility of gaps in genetic information, failing to account for all relevant genetic factors influencing smoking behaviour and glaucoma risk. Additionally, MR inferences depend on instrument validity and phenotype definitions, and results can be sensitive to pleiotropy and measurement error. For instance, it has also recently been reported that cigarette smoking increases the corneal biomechanical resistance to deformation, with little evidence to support a relationship with glaucoma [76]. This raises the possibility that discordant “protective” signals may reflect methodological artefact or complex trait pathways rather than a true protective effect, which may help explain inconsistency across studies and with our review.

### Clinical implications of smoking

While smoking does not appear to increase the risk of developing POAG, it significantly elevates the risk of other ocular diseases. Smokers are significantly more likely to develop age-related macular degermation (ARMD) compared to non-smokers [77]. Additionally, current smokers are at a significantly greater risk of developing cataracts, which then necessitates surgical intervention [16].

For individuals already diagnosed with glaucoma, continuing to smoke poses compounded risks. The potential development of ARMD would result in both peripheral vision loss from glaucoma and central vision loss from ARMD, leading to a more profound visual impairment. Therefore, smoking cessation advice should be offered to these patients as it has unequivocal cost effectiveness on the impact of ARMD [78]. Moreover, elevated IOP associated with glaucoma can increase the complexity and risks associated with cataract surgery [79, 80]. It remains important to advise patients to stop smoking, but in those with an established glaucoma diagnosis, this comprehensive meta-analysis spanning 75 + years of 26 studies and 289,930 subjects, has shown with a high degree of certainty the lack of an association with smoking. It remains imperative to cease smoking to mitigate the risk of potential vascular, systemic or ocular diseases that could further compromise their visual function.

## Limitations

We observed varying levels of heterogeneity contributing to the variability among our results. This heterogeneity arose from multiple sources. Clinical heterogeneity resulted from differences in participant characteristics, such as age, sex, ethnicity, and the outcomes measured or how they were defined. Methodological heterogeneity stemmed from variations in study design, differences in study quality including blinding and allocation concealment, and differences in the tools or instruments used to measure outcomes. For the pooled analysis of combined smokers, between-study heterogeneity was high (I^2^ = 79%), and this was driven primarily by heterogeneity in smoking exposure definitions (e.g., current vs ever/lifetime smoking; differing thresholds for intensity or duration such as pack-years; and inconsistent criteria for “former” smoking) and by variability in covariate adjustment across studies (e.g., age, sex, ethnicity, IOP, CCT, blood pressure/ocular perfusion pressure, diabetes, hypertension). These definitional and analytical differences plausibly inflate dispersion in effect sizes and limit the comparability of pooled estimates. Importantly, inconsistent and frequently self-reported exposure definitions are a major source of potential misclassification: broad or undefined categories (e.g., “current” or “past” smoking without recency, duration, or intensity thresholds) may mix non-comparable exposure constructs across studies. This type of non-differential misclassification would be expected to bias associations towards the null, potentially obscuring a true dose–response relationship, while simultaneously increasing between-study heterogeneity. Accordingly, where I^2^ was high (notably the ‘current and past smokers combined’ comparison, based on six studies), pooled estimates should be interpreted cautiously. Study-design subgroup analyses (reported in Figs. 2, 3, 4, 5) demonstrate that some strata contain few studies and yield less stable estimates, which is reflected in our certainty ratings (GRADE very low for current and past smokers combined).

One of these factors was the definitions of current smoking and past smoking (Table 1, 2). Given the time span covered by included studies, smoking behaviours and cigarette composition have also changed over time [81], which may contribute to temporal and contextual heterogeneity. However, because most studies did not report cigarette type or standardised exposure intensity measures in a comparable way, we cannot directly link historical changes in cigarette design to differences in pooled effect estimates. Statistical heterogeneity was evident in the variation of effect sizes reported across studies and the different statistical methods employed for data analysis. Additionally, contextual heterogeneity occurred due to differences in study settings, such as primary versus secondary care, geographical locations, and the time periods during which the studies were conducted.

In view of this, sensitivity analyses excluding studies at high overall risk of bias yielded effect estimates that were directionally and quantitatively consistent with the primary analyses across all smoking categories, confirming that the observed lack of association between smoking and primary open angle glaucoma is not driven by inclusion of lower quality studies.

## Conclusion

In conclusion, no causal or protective relationship between smoking and POAG was observed. There is significant heterogeneity between studies. Although, smoking may not be related to POAG, there is evidence that smoking is associated with the development of ocular conditions including but not limited to, retinal vascular occlusions, age-related macular degeneration, cataract and thyroid eye disease [82–84]. Therefore, it remains important that patients are counselled to stop smoking, due to its negative effects on visual function and general health.

## Data Availability

No datasets were generated or analysed during the current study.

## Abbreviations

POAG: Primary open-angle glaucoma
RCT: Randomised controlled trial
OR: Odds ratio
RR: Risk ratio/relative risk
HR: Hazard ratio
95% CI: 95% Confidence intervals
ROB: Risk of bias
